# Acute malnutrition and its contributing factors among children under-five years in rural kebeles of Shashemene Oromia, Ethiopia

**DOI:** 10.3389/fnut.2022.1053928

**Published:** 2022-12-21

**Authors:** Nigusie Shifera, Aschalew Endale, Degfachew Debela, Tewodros Yosef

**Affiliations:** ^1^School of Public Health, College of Medicine and Health Sciences, Mizan Tepi University, Mizan Teferi, Ethiopia; ^2^Public Health Department, Arsi University, Asella, Ethiopia; ^3^Public Health Emergency Department, Ethiopian Public Health Institutions, Addis Ababa, Ethiopia

**Keywords:** prevalence, acute malnutrition, children, rural, Ethiopia

## Abstract

**Introduction:**

Globally, more than 52 million under-five years old were wasted; One-third of these children live in Africa. Ethiopia is the seventh country among the ten top countries in which acute malnutrition (AM) is concentrated and currently 10% of under-five children are wasted. Even though Ethiopia has implemented a variety of nutritional interventions, acute malnutrition is still prevalent and spreading at an alarming rate. Therefore, this study aimed to assess the prevalence of acute malnutrition and its contributing factors among children under-five years of age.

**Materials and methods:**

A community-based cross-sectional study was conducted from July 1 to 30, 2018 among 12 randomly selected kebeles. The sample sizes were proportionally allocated to the selected kebeles. A total of 457 mothers/caretakers of under-five children were interviewed using pre-tested structured questionnaires and anthropometric measurements of the children were taken using standard procedures. EPI data version 4.2 was used for data entry and Statistical Package for the Social Sciences (SPSS) Version 21 was used for statistical analysis. The World Health Organization (WHO) Anthro software was used to convert nutritional data indices. Binary logistic regression was used to determine the association between dependent and independent variables. The level of significance was declared at a *P*-value < 0.05.

**Results:**

The prevalence of acute malnutrition is 19.91% (95%CI; 16.24%, 23.57%) among under-five children. Factors contributing to acute malnutrition were mothers with no antenatal care (ANC) visits [adjusted odds ratio (AOR) = 2.26, 95% CI 1.14–4.46], mothers who had no autonomy in decision-making (AOR = 2.42, 95% CI 1.42–4.12), children with diarrheal disease in the last 2 weeks preceding the survey (AOR = 2.07, 95% CI 1.19–3.59), and not feeding colostrum (AOR = 1.99, 95% CI 1.07–3.71).

**Conclusion and recommendation:**

The prevalence of acute malnutrition is high as compared to other findings in Ethiopia. Moreover, decision-making power, not feeding colostrum, no ANC visit, and a child's history of diarrhea were independent determinants of acute malnutrition. Therefore, the local health department and health extension workers should consider imparting health education for women on nutritional counseling and timely treatment for children with diarrhea. Empowering women's decision-making is also a key element in addressing wasting among under-five children.

## Introduction

Malnutrition is the lack of the right kinds of food and nutrients needed by the body for appropriate growth and development. It includes undernutrition and overnutrition. Stunting, wasting, and being underweight are classified as among Anthropometric indicators commonly used to measure malnutrition in a population of under-five children (1). Acute malnutrition (AM) is classified into severe acute malnutrition (SAM) and moderate acute malnutrition (MAM) according to the degree of wasting and the presence of edema (2).

Acute malnutrition develops as a result of rapid weight loss or a failure to gain weight. In children, it is assessed via the nutritional index of Weight-for-height, Mid-Upper Arm Circumference (MUAC), and edema is used to decide acute malnourishment. If a child's weight for height measurement is <70% of the normal range for his age, or when the child's MUAC is <11 cm, then the child would be diagnosed with severe acute malnutrition. The presence of one criterion is sufficient to categorize a child as severely malnourished (3). Successful management of severe acute malnutrition requires a medical intervention combined with a community-based approach to help with the timely detection and appropriate provision of treatment (4).

Globally in 2015 about 480,000 under-five children continue to die every month. Undernutrition causes deaths in 53% of children under-five years, acute malnutrition is responsible for more than 28% (5), and around one million children under the age of five years die every year from severe acute malnutrition (6). Globally, about 52 million under-five years old were wasted and among this around 32% (16 million) were severely wasted (7), one-third of these children live in Africa, especially in Sub-Saharan Africa (SSA) (8).

In Ethiopia; the 2016 Ethiopian Demographic and Health Survey (EDHS) revealed that there has been an improvement in the nutritional status of children. The percentage of stunting fell from 40% in 2014 to 38% in 2016. Similarly, the percentage of underweight children declined from 25% to 24%, but the prevalence of wasting increased from 9% to 10%. Furthermore, the prevalence of wasting is different from region to region for instance in Oromia's national regional state, the percentage of children who were wasted is 10.6% and in the Somali regional state was 22.7% (9). Recent studies conducted in Kamashi district and Seqota district, Amhara region, Ethiopia revealed acute undernutrition accounted for 4.3% and 8%, respectively (10, 11).

Currently, there are different nutritional interventional programs and activities being held by the government concerned with policy-making and implementation programs like the Community based Nutrition Program (CBN), Essential Nutrition Action (ENA), Community Health Day (CHD), Infant and Young Child Feeding program (IYCF), Community Management of Acute Malnutrition (CMAM). In line with the government's role, there are also different local and international NGOs working in Ethiopia for the overall achievement of the sustainable development goals (SDG). Despite the entire effort, the trend of decline in the undernutrition problem is not satisfactory (12–14).

Even though Ethiopia has been implementing different nutritional programs, wasting is still the top leading public health problem for under-five years children (15, 16). Ethiopia is still the seventh country among the ten top countries in which wasting (acute malnutrition) is concentrated. Even though, the previous studies pointed out the prevalence of wasting, most of these studies do not identify the key determinants of wasting among the rural kebele community (17). Therefore, this study aimed to identify the prevalence of acute malnutrition and its contributing factors among children under-five years of age in rural kebeles of Shashemene district, Oromia region, Ethiopia.

## Materials and methods

### Study setting, design, and period

A community-based cross-sectional study was conducted from July 1 to 30, 2018 in the Shashemene district of Oromia, Ethiopia. The district is located 250 km far from Addis Ababa; the capital city of Ethiopia. The district has 38 rural administrative kebeles with 265,109 total populations in 55,231 households with males to female ratio of 1:1.02. The projected number of under-five children is 43,557 projected from the 2007 national population census (18). Regarding health infrastructures, there are eight health centers, three private clinics, and thirty-eight health posts. The majority of the population was engaged in agricultural activities in the district and the main crops/products were Maize, Teff, and Sorghum, fruits and vegetables, and animal husbandry.

### Populations

All children under-five years living in rural kebeles of sahshemene zone were the source population. Those selected children who lived in the selected households during the study period were the study population. Physically disabled children, ages below-five years, and critically ill children were excluded from the study.

### Sample size determination and sampling procedures

The sample size was determined using single population proportion formula by considering an assumption of 95% confidence level, 5% margin of error, and 23.6% prevalence of acute malnutrition from a previous study done in Hawassa Zuria district, Southern Ethiopia (8).

The following assumption was used.


$$\begin{array}{l} n={\left(Z_{\alpha /2}\right)}^{2^{*}}{p\;\left(1-p\right)}^{2}/d^{2}=\frac{\left(0.764\right)\;\left(0.236\right){\left(1.96\right)\;}^{2}}{{\left(0.05\right)}^{2}}=\;277. \end{array}$$

After considering a design effect of 1.5 and 10% non-response rate compensation; the final sample size became **457**.

### Sampling procedures

A multi-stage sampling technique was applied. From the 38 rural kebeles in the Shashemene district, 12 kebeles were selected using simple random sampling. From each kebele, three gotes were again randomly selected. The total sample size was proportionally distributed to each gote based on their population size. A maximum of one child aged younger than five years was included randomly in each selected household.

### Study variables

The dependent variable was acute malnutrition (wasting). The independent variables were socio-demographic characteristics (maternal/parental education, maternal/parental occupation, family size), child and health-related characteristics (sex, age, morbidity status), child caring practices/nutritional care and practice [exclusive breastfeeding practice, complementary feeding (CF) practice, duration of breastfeeding, immunization status, feeding of sick child] maternal characteristics/obstetric factors (number of children ever born, maternal autonomy in decision making, use of extra food during pregnancy or lactation), environmental health condition (access to latrine, access to safe water, access to health facilities).

### Operational definitions

**Acute malnutrition (wasting)**: a child's weight for height measurement is <-2 SD or bilateral pitting edema.

**Bilateral pitting edema**: pitting edema of the feet verified when thumb pressure applied on top of both feet for 3 s leaves a pit (indentation) in the foot after the thumb is lifted (19).

**Critically ill**: a child who has chronic diseases like severe pneumonia, TB, CHF, DM, and HIV/AIDS.

**The dietary diversity score (DDS)** is the sum of the total number of food groups consumed over 24 h before the data collection (20).

**Healthcare-seeking behavior:** healthcare-seeking behavior by the caregiver of the child within 24 h of the onset of symptoms.

**Immunization status:** either vaccinated or not, if possible, look at the vaccination card or ask the mother (caregiver).

**Morbidly status of the child**: morbidity status in the last 2 weeks before the study period (fever, cough, diarrhea).

**Complementary foods** are foods that are required by the child, after 6 months of age, in addition to sustained breastfeeding.

### Data collection procedures (instruments, personnel, measurements)

For face-to-face interviews, a pre-tested and standardized questionnaire with questions adopted and modified from different literature reviews was used to collect data. Trained data collectors were assigned to all gote to collect the data. The vaccination status of the children was assessed by looking at the vaccination card or asking the mother (caretaker).

Anthropometric data were collected through the measurement of the length/height and weight of all children. A Salter hanging spring scale with graduations of 0.1 kg and a capacity of 25 kg were used for measuring the weight of the children aged 6–23 month and a beam scale for children over 24 months of age were used for measuring weight with minimum clothing and no shoes to the nearest 0.1 kg. The recumbent length measurement was taken for children under 2 years of age, then subtract 0.7 cm to convert the result to height while for children above 2 years stature was measured in a standing position in centimeters to the nearest of 0.1 cm (3). Edema of the feet was diagnosed if a bilateral depression (pitting) remained after the pressure was released (19).

Child dietary diversity score was calculated by summing a total of seven food groups: (1) grains, roots, and tubers; (2) vitamin a-rich fruits and vegetables; (3) dairy products; (4) flesh foods (meat, fish, poultry, and liver or organ meat); (5) other fruits and vegetables; (6) egg; (7) legumes and nuts; consumed over the reference period (24 h before the data collection). For example, if one child eats from each food group, his/her DDS will be 7 (20).

### Data quality assurance

The questionnaire was translated into the local language, Afan Oromo, for the fieldwork and back to English to check its consistency among three health workers who are fluent speakers of Afan Oromo and English. Besides, it was pre-tested in Shalla district in 5% of the sample size. Twelve diploma nurses for data collection and four BSc nurses for supervision were trained for 2 days. The training covered in the questionnaire and the importance of disclosing the possible benefits and purpose of the study to the study participants before starting data collection. The data collection was supervised by the principal investigator. Every questionnaire was supervised and reviewed for completeness and logical consistency. The completeness of the questionnaire was also checked before data entry. Validation of instruments, measurements, and random auditing was done daily. The double data entry would be used to ensure data quality.

### Data processing and analysis

EPI info version 7 software was used for data entry (21) and cleaning and then the data were exported to Statistical Package for the Social Sciences (SPSS) Version 21 for further processing and analysis (22). World Health Organization (WHO) Anthro software was used to convert nutritional data indices from anthropometric measurements into Z-scores. A binary logistic regression model was used to compute the association between each independent variable and dependent variable. Independent variables that show association with outcome during bivariate analysis at a *P*-value <0.25 were included in multivariable analysis to control for potential confounders. Descriptive analysis was used to describe the percentages and number of distributions of the respondents by socio-demographic characteristics and other relevant variables in the study.

Homer–Lemeshow goodness-of-fit was used to test for the model fitness and a backward step-wise (likelihood ratio) method was also conducted. Adjusted odds ratio (AOR) along with a 95% confidence interval (CI) was estimated to assess the strength of the association and a *P*-value < 0.05 was considered to declare the statistical significance in the multivariable analysis in this study.

### Ethical consideration

Ethical clearance was obtained from the Institutional Review Board at the College of Health Sciences of Arsi University. In addition, a permission letter from the Shashemene District Health Office was obtained before field activities started, and written informed consent was obtained from the parents/caretakers of the study subjects after explaining the study objective and procedures.

## Results

### Socio-demographic characteristics

A total of 457 under-five children participated with a response rate of 100%. The mean age (SD) of the children and mothers was 25 (3.4) months and 26.9 (4.3) years, respectively. A household headed by a male or a married person accounted for more than 90% of the respondents [433 (94.75%) and 414 (90.59%)], respectively. 14 (90.60%) of the mothers were Oromo, while 366 (80.09%) of the respondents identified as Muslims. In a similar proportion, 240 (52.52%) of the caregivers had formal education and were autonomous in households' decision-making (Table 1).

**Table 1:** Socio-demographic characteristics of study participants in Shashemene district, Oromia region, Ethiopia, July 2018 (457).

**Variables**	**Categories**	**Total**	

		**Frequency**	**%**
Head of the HH	Male	433	94.75
	Female	24	5.25
Ethnicity	Oromo	414	90.60
	Wolayita	30	6.56
	Others^*^	13	2.84
Religion	Muslim	366	80.09
	Protestant	42	9.19
	Orthodox	32	7.00
	Catholic	17	3.72
Land ownership	Yes	384	84.03
	No	73	15.97
Maternal literacy	Yes	240	52.52
	No	217	47.48
Paternal literacy	Yes	275	60.18
	No	182	39.82
Area of agricultural land	<1 hectare	16	4.17
	1–2 hectare	342	89.06
	>3 hectare	26	6.77
Occupation of the mother	Housewife	421	92.12
	Merchant	27	5.91
	Other^**^	9	1.97
Occupation of the husband	Farmer	374	81.84
	Merchant	60	13.13
	Other^**^	23	5.03
Family size	≤ 5	314	68.71
	>5	143	31.29
Maternal autonomy	Yes	240	52.52
	No	217	47.48
Marital status	Married	414	90.59
	Divorced	26	5.69
	Other^***^	17	3.72

* Amhara, Wolayita, Sidama.

** Employee, daily laborer.

*** Widowed, separated, single.

### Maternal and child characteristics

Of the total children, 206 (45.08%) were delivered at home and 251 (54.92%) children were delivered at health facilities. Concerning vaccination status and vitamin A supplementation, 432 (94.53%) of children were vaccinated and 181 (41.51%) of children were supplemented with vitamin A, respectively. Among the surveyed 457 mothers 432 (94.53%) of mothers/caregivers of children were in the age group <35 years and 395 (86.43%) mothers of under-five children practiced at least one antenatal care (ANC) visit. While 135 (29.54%) and 118 (25.82%) mothers did not take extra meals during pregnancy and lactation, respectively (Table 2).

**Table 2:** Maternal and child characteristics of study participants in Shashemene district, Oromia region, Ethiopia, July 2018 (457).

**Variables**	**Categories**	**Total**	

		**Frequency**	**%**
Age of the mothers/caregivers (years)	<35	432	94.53
	≥35	25	5.47
Maternal extra meal during the last pregnancy	0	135	29.54
	1	234	51.20
	2	88	19.26
Maternal extra meal during the last lactating	0	118	25.82
	1	242	52.95
	2	97	21.23
ANC visit	Yes	395	86.43
	No	62	13.57
Age of the mothers at first birth	≤ 20	384	84.03
	>20	73	15.97
Distance from the HH to the HF (min)	<30	317	69.37
	≥30	140	30.63
Age of the child (months)	0–5	21	4.60
	6–23	203	44.42
	24–59	233	50.98
Sex of the child	Male	213	46.61
	Female	244	53.39
Child received deworming	Yes	98	43.95
	No	125	56.05
The child received vitamin A	Yes	181	41.51
	No	255	58.49
Type of birth	Single	440	96.28
	Multiple/Twins	17	3.72
Child vaccination status	Yes	432	94.53
	No	25	5.47
Place of birth	Home	206	45.08
	HI	251	54.92
History of diarrhea in the last 2 weeks	Yes	111	24.29
	No	346	75.71
History of febrile illness in the last 2 weeks	Yes	28	6.13
	No	429	93.87

### Child caring practice

Slightly above two-thirds 320 (70.02%) of children had breastfeeding that was initiated within 1 h after birth and about 90 (19.69%) of children took pre-lacteal feeding. About 281 (61.49%) mothers exclusively breastfeed their children for the first 6 months. Three-fourth 323 (74.08%) of the children started complementary feeding in addition to breastfeeding at the age of 6 months while 111 (25.46%) of the children started complementary feeding. Spoon feeding was practiced in 240 (55.04%) children while 147 (33.72%) and 49 (11.24%) used bottle and cup feeding, respectively. In addition, 315 (72.25%) of children were consume diversified food groups <4 within 24 h while 121 (27.75%) of children were consume diversified food groups ≥4 within 24 h (Table 3).

**Table 3:** Child caring practices of the study population in Shashemene district, Oromia region, Ethiopia, July 2018 (457).

**Variables**	**Categories**	**Total**	

		**Frequency**	**%**
Duration of breastfeeding	<6 months	19	4.16
	Up to 12 months	54	11.81
	For 24 months	296	64.77
	More than 24 months	88	19.26
DDS	<4	315	72.25
	≥4	121	27.75
Time of breastfeeding initiation	Within the first hour	320	70.02
	After the first hour of delivery	137	29.98
Child deprive colostrum	Yes	81	17.72
	No	376	82.28
EBF of the child during the first 6 months	Yes	281	61.49
	No	176	38.51
Age of the child while starting CF	<6 months	111	25.46
	At 6 months	323	74.08
	6–12 months	2	0.46
Method of feeding	Bottle	147	33.72
	Cup	49	11.24
	Spoon	240	55.04
Pre-lacteal feeding	Yes	90	19.69
	No	367	80.21
Frequency of breastfeeding	4–7	155	33.92
	≥8	302	66.08
Increase the pattern of feeding for a sick child	Yes	222	48.58
	No	235	51.42

### Environmental factors

About 399 (87.30%) households got water from piped outside the compound. The average water utilized per household was 35 L per day and the average round trip to fetch water from the source of water was 37.24 min. Concerning household domestic liquid and solid waste disposal, 274 (59.96%) and 234 (51.20%) of the households disposed of domestic liquid and solid waste in open fields, respectively, and latrine was not available in 75 (16.41%) households. Regarding the residence house, 269 (58.86%) households had thatched homes while 188 (41.14%) were corrugated iron sheets (Table 4).

**Table 4:** Environmental characteristics of the study population in Shashemene district, Oromia region, Ethiopia, July 2018 (457).

**Variables**	**Categories**	**Total**	

		**Frequency**	**%**
Latrine availability	Yes	382	83.59
	No	75	16.41
Domestic liquid waste disposal	Open field	274	59.96
	Pit	183	40.04
HH domestic solid waste disposal	Open field	234	51.20
	Pit	133	29.10
	Burning	10	2.20
	Composting	80	17.50
Type of residence house	Thatched	269	58.86
	Corrugated iron sheet	188	41.14
Kitchen site	Inside residential house	140	30.63
	Separated	317	69.37
Live together with animals	Yes	127	27.80
	No	330	72.20
Source of drinking water	Piped outside the compound	399	87.30
	Covered well	21	4.60
	Protected spring	35	7.66
	Open well	2	0.44
Take to HI when the child is sick	Within 24 h	235	51.42
	More than 24 h	222	48.58

### Prevalence of acute malnutrition

**Figure 1:**
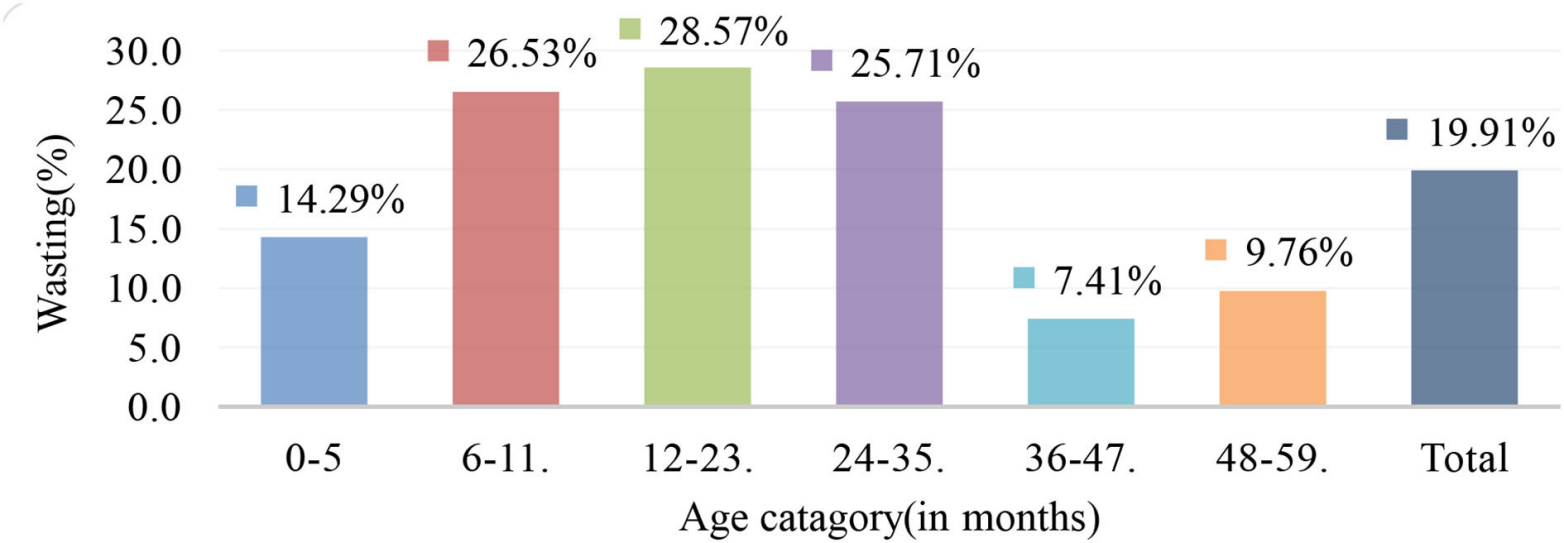
Prevalence of acute malnutrition (wasting) by age category among under-five children in Shashemene district, Oromia region, Ethiopia, July 2018.

The prevalence of acute malnutrition among under-five children was 91 (19.91%) with (95% CI; 16.24%, 23.57%). In children aged 0–5, 6–11, 12–23, 24–35, and 36–47 months, the prevalence of wasting was 3 (14.29%), 26 (26.53%), 30 (28.57%), 18 (25.71%), 6 (7.31%), and 8 (9.76%), respectively (Figure 1).

### Factors associated with acute malnutrition (wasting)

In bivariate analysis, parental education, maternal autonomy in decision making, squeezed out, and throwing colostrums, ANC visit, history of diarrhea in the last 2 weeks, history of febrile disease in the last 2 weeks, age of mother at first birth, place of birth, maternal education, and pre lacteal feeding were the variables that showed an association with the outcome variable at the bivariate analysis with *P*-value <0.25 were entered into the final multivariable logistic regression to control potential confounders.

On multivariable logistic regression, maternal ANC visits were significant predictors determinants for acute malnutrition, children whose mothers were not on ANC visits at least once were two times more likely to be acutely malnourished than those children whose mothers were on ANC visits at least once (AOR = 2.26, 95% CI 1.14–4.46). The odds of developing wasting were two times higher among children whose mother has not autonomous in decision-making than those whose mother has autonomous in decision-making (AOR = 2.42, 95% CI 1.42–4.12).

The morbidity status of the child by diarrheal disease in the last 2 weeks preceding the survey was also seen as one of the determinants of acute malnutrition in under-five children. Acute malnutrition was two times more likely to occur in children who had diarrhea before 2 weeks than those children who had no diarrhea within 2 weeks (AOR = 2.07, 95% CI 1.19–3.59). Although children who had a fever in the last 2 weeks preceding the survey shows a significant association in bi-variable analysis, no association was observed between children who had a fever in the last 2 weeks preceding the survey and wasting in multivariable analysis. Similarly, children whose mothers had squeezed out colostrum were two times more likely to be wasted than under-five children whose mothers had no squeeze-out colostrum (AOR = 1.99, 95% CI 1.07–3.71) (Table 5).

**Table 5:** Factors associated with acute malnutrition (wasting) among children under-five years, of Shashemene district, Oromia region, Ethiopia, and July 2018 (457).

**Variable**	**AM (wasting)**		**COR (95%, CI)**	**AOR (95%, CI)**	***P*-values for AOR**

	**Yes**	**No**			
**Paternal literacy**					
Yes	43	232	1		
No	48	134	1.93 (1.19–3.03)	1.36 (0.77–1.98)	0.453
**Maternal autonomy**					
Yes	39	201	1	1	
No	52	165	1.62 (1.02–2.58)	2.42 (1.42–4.12)	**0.001**
**ANC visit**					
Yes	71	324	1	1	
No	20	42	2.17 (1.20–33.92)	2.26 (1.14–4.46)	**0.019**
**History of diarrhea in the last 2 weeks**					
Yes	30	81	1.73 (1.05–2.56)	2.07 (1.19–3.59)	**0.010**
No	61	285	1	1	
**History of febrile illness in the last 2 weeks**					
Yes	10	18	2.39 (1.06–5.37)	1.94 (0.99–3.74)	0.067
No	81	348	1		
**Child deprive colostrum**					
Yes	28	53	2.62 (1.54–4.47)	1.99 (1.07–3.71)	**0.029**
No	63	313	1	1	
**Age of the mother at first birth**					
≤ 20	68	316	1		
>20	23	50	2.14 (1.22–3.74)	1.93 (0.95–2.74)	0.216
**Place of birth**					
Home	50	156	1.64 (1.03–2.61)	1.42 (0.86–2.56)	0.432
HI	41	210	1	1	
**Maternal illiteracy**					
Yes	37	203	1	1	
No	54	163	1.82 (1.14–2.89)	1.67 (0.87–2.95)	0.322
**Pre-lacteal feeding**					
Yes	31	59	2.69 (1.61–4.50)		
No	60	307	1		
**Maternal extra meals during lactating**					
0	27	91	2.90 (1.29–6.52)	2.14 (0.97–3.74)	0.065
1	55	187	2.88 (1.36–6.08)	2.04 (0.88–2.74)	0.087
2	9	88	1		

## Discussion

In this study, the prevalence of acute malnutrition (wasting) is 19.91% (95%CI; 16.24%, 23.57%) and maternal autonomy in decision making, squeezing out, and throwing colostrum, ANC visit, history of diarrhea in the last 2 weeks, were the independent factors associated with acute malnutrition.

The above-stated prevalence of acute malnutrition (wasting) was higher as compared with 14.2% in Kwara State, Nigeria (23), and 16.5% in Nakaseke and Nakasongola districts, Uganda (24) from African countries. Moreover, the study is higher than 13.4% in the Bule Hora district (25), 16.7% in Hidabu Abote, North Shewa (26), and 9% in Damot Gale (27), studies in Ethiopia. This difference might be due to the variation in the study methodology used and the great variation between the rural and urban communities. Unlike this study, the previous studies mentioned above were conducted in the urban community.

Although present study showed that the prevalence of wasting was high in the district in comparison with the regional (10.6%) and national prevalence (11%) reported by EDHS 2016 (9). The discrepancy might be due to the sample size compared to that of national data. This finding was consistent with 23.6% in Hawassa Zuria district, Southern Ethiopia (28). This similarity may be due to socioeconomic, study design, and sample size. However, this finding was lower than 42.3% in Dolo Ado district, Somali region, Ethiopia (29). This may be due to Dolo Ado district being highly affected by drought and the community being pastoralists. Moreover, the finding was lower than 25% in Christ specialist hospital, Ogui, Enuu, Nigeria, and higher than 14.8% at the University of Nairobi, Kenya (30, 31). This difference might be due to the study design, sociodemographic characteristics, lifestyle, and cultural variation.

Maternal ANC visit was a significant determinant of acute malnutrition. Children whose mothers had no ANC visits were two times more likely to develop acute malnourished than those children whose mother has ANC visits. Since the overall aim of ANC is to produce a healthy mother and baby at the end of pregnancy, taking antenatal visits may help the mother and child to have better health and knowledge of the child-caring practice. This finding was consistent with a study conducted in Damot Gale district, South Ethiopia (27), which revealed that children whose mothers attended ANC were associated with acute malnutrition. However, another study conducted in Shone district, Hadiya zone, SNNPR (32), showed that children whose mothers haven't had antenatal care follow-up are associated with acute malnutrition of the children.

Children whose mothers were not autonomous in decision-making were two times more likely to be wasted than those children whose mothers were autonomous in decision-making. This result was consistent with studies conducted in Shashogo district and Machakel district (33, 34), and studies in Ethiopia. Such findings will require women's autonomy to participate in the decision-making process of the household equally with their husbands. Nowadays the policy of government also supports empowering women, and women's education and the increasing influence of women have a significant impact on the health of the family and community.

Children with diarrhea preceding 2 weeks before the onset of acute malnutrition are significantly associated with acute malnutrition (wasting) children. This can be due to excessive loss of fluids and electrolytes, loss of appetite, or lack of absorption of food in the intestine due to high motility of the intestine during diarrhea episodes (5). This finding was supported by studies done by Shibru and Tesfaye and Bantamen et al. (32, 33).

Squeezing out of first milk was significantly associated with acute malnutrition in under-five children. Children deprived of colostrum were two times more likely wasted than those children who have not deprived of colostrum. This result was consistent with a case-control study conducted in the Machakel district revealed that squeezing out of first milk showed a significant association with malnutrition (33). Exclusive breastfeeding is recommended because breast milk (colostrum) is containing necessary nutrients in the first few months of life. In addition, the mother's antibodies in breast milk provide the infant with immunity to disease. Early supplementation exposes infants to pathogens and thus increases their risk of infection, especially diarrheal disease and it decreases infants' intake of breast milk and therefore suckling, which in turn reduces breast milk production (35).

## Strengths and limitations

The study considers a high-risk population (children 0–5 months of age group), using World Health Organization (WHO) Anthro software and close supervision throughout the field activities were the strength of the study. However, recall bias for some variables (for example DDS, frequency of breastfeeding, complementary feeding) is a limitation of the study.

## Conclusion and recommendation

The prevalence rate of acute malnutrition (wasting) among under-five children in the district was high. Antenatal care visits, decision-making, squeezing out and throwing colostrum, and children who had diarrheal disease were the independent predictors of acute malnutrition. Therefore, the local health department and health extension workers should consider imparting health education for women on nutritional counseling and timely treatment for children with diarrhea. Moreover, the health departments try to consider the effects of these factors (women's decision-making, benefits of colostrum, diarrheal disease, and ANC) while proving services on nutrition.

## Data availability statement

The original contributions presented in the study are included in the article/supplementary material, further inquiries can be directed to the corresponding author/s.

## Ethics statement

The studies involving human participants were reviewed and approved by Mizan Tepi University. The patients/participants provided their written informed consent to participate in this study.

## Author contributions

All authors wrote the protocol, participated in data collection, analyzed the data, and wrote the manuscript. All authors contributed to the article and approved the submitted version.
